# The Frequency of Periorbital Hyperpigmentation Risk Factors

**DOI:** 10.1111/jocd.70036

**Published:** 2025-02-09

**Authors:** Fateme Heidari, Mohammad Ebrahim Zadeh, Maryam Haji Abole Zadeh, Nasim Namiranian

**Affiliations:** ^1^ Department of Medicine, Yazd Branch Islamic Azad University Yazd Iran; ^2^ Department of Dermatology Shahid Sadoughi University of Medical Sciences Yazd Iran; ^3^ Department of Medicine Shahid Sadoughi University of Medical Sciences Yazd Iran; ^4^ Diabetes Research Center Shahid Sadoughi University of Medical Sciences Yazd Iran

**Keywords:** dark circles, infraorbital pigmentation, pigmentation, preorbital hyperpigmentation

## Abstract

**Background:**

Periorbital hyperpigmentation (POH) is a common complaint worldwide. Although this cosmetic condition is not a medical concern, it can impact emotional well‐being. Few investigations have evaluated the association of various factors with POH, which is contradictory in some ways.

**Aim:**

The present study is conducted to elucidate the accurate impact of risk factors on the severity of POH.

**Patients/Methods:**

This cross‐sectional study was performed on 116 patients attending a skin Outpatient Department in Iran from March to August 2022. A complete physical examination divided patients into mild, moderate, severe, and very severe classes. Valid questionnaires were filled out by participants about their demographic data, underlying diseases, and their habits. The chi‐square and linear regression tests were done. All data were analyzed using SPSS version 24.

**Result:**

Among demographic factors, positive family history had a significant association with POH. There was a significant association between POH and stress, prolonged exposure to sun or television, and taking hormonal pills. Surprisingly, sleep quality was not significantly related to POH. In addition, there was a relationship between POH and some underlying diseases.

**Dissuasion:**

The present study agreed with others about the significant impact of factors like family history, prolonged sun exposure, stress, and anemia. The current investigation elucidated doubts about the insignificant effect of sleep quality on the severity of POH. In contrast to previous studies, a remarkable relationship between POH and atopy was not revealed.

**Conclusion:**

POH is an undesirable condition. Two key factors associated with this cosmetic problem are rubbing eyes and prolonged exposure to television.

## 1 Introduction

Periorbital hyperpigmentation (POH), known as “dark circles,” is a common complaint in patients referred to the outpatient department of dermatology [1]. This significant cosmetic problem usually presents as symmetric pigmentation over the bilateral periorbital area that affects either the upper or lower eyelids or both [2]. It may also spread to the eyebrows, temporal region, malar region, and neighboring regions [3].

This condition may occur across all age groups, both sexes, and all races [3]. The highest phototypes are usually the most affected population [4]. In an investigation conducted in India, the prevalence of POH reported 30.8%, and most subjects were women [5]. Although prevalence studies are deficient, the significance of POH is reflected in the cosmetic industry, where under‐eye concealers make up a large portion of the market [6]. Cosmetic complaints that are not health threatening but can impact emotional well‐being are receiving more attention. POH is an important cosmetic concern, particularly for women, that can give the face a tired, stressed, and less youthful look [3, 7]. Various cosmetic treatments, surgery, and lasers are used to improve this condition [8].

The etiology of POH is complex and multifactorial [7, 9]. The influence of lifestyle factors on the prevalence and severity of POH has been evaluated; however, some misconceptions may persist [8]. For example, it is demonstrated in some studies that lack of sleep can lead to POH or worsen existing POH [10, 11]. In contrast, inadequate sleep is indicated to be related to POH by few investigations [12]. Obviously, this variable is discussed as controversial [5, 13]. Despite the high prevalence of POH, limited studies have tried to detect its causes. This comprehensive and detailed study is conducted to determine the impact of risk factors on the severity of POH.

## 2 Materials and Methods

This cross‐sectional study was performed on all subjects who referred to the skin clinic affiliated with Shahid Sadoughi University of Medical Science, Yazd, Iran, from March to August 2022. A total of 116 patients with POH of both genders and over 18 years of age were included in the study. Pregnant and lactating females and those with ocular pathology and skin disease likely to interfere with the measured parameters were excluded.

All evaluations were achieved in a controlled environment (under standardized lighting, 297.15 ± 2 K and 40% ± 10% relative humidity) on skin that had not been treated with product or makeup since the evening before the visit. Patients were recommended to rest in the examination room for 30 min before assessments to ensure stable blood flow and skin temperature.

Then, a careful physical examination was conducted by an experienced physician to detect hyperpigmented areas in the lower, upper, or both eyelids and their extension beyond the periorbital region. An eyelid stretch test was done to rule out the shadow effect. Darkness around the eyes was divided into mild, moderate, severe, and very severe classes.

A detailed history of age, gender, family history of POH, and atopic history was taken in a standard form. Additionally, incorrect habits of participants were entered in standard form, including insomnia, stress, usage of prescription and sunglasses, usage of hormonal pills and oral contraceptive pills (OCP), prolonged exposure to bright screens (computer, cell phone, and television), prolonged exposure to the sun (more than 5 h a day), rubbing eyes, and frequent use of cosmetics and hygiene products (daily use of makeup substances).

The frequency and type of underlying diseases, including anemia, diabetes, hypertension, thyroid disorders, gastrointestinal diseases, hepatobiliary diseases, renal diseases, and others, were also recorded to find any associations. The findings were recorded on standard forms. Additionally, PSS and ISI questionnaires have been used to record stress and sleep disorder variables.

The Perceived Stress Scale (PSS) is one of the most usable psychological tools for determining stress perception. The scale includes 14 direct questions about present levels of experienced stress. The queries in the PSS ask about thoughts and feelings during the last month. This 5‐point Likert scale rates each item (never = 0, almost never = 1, sometimes = 2. fairly often = 3, very often = 4). PSS scores are collected by reversing the rates on the seven positive items and then collecting all 14 items. Items 5, 6, 7, 8, 9, 10, and 13 are the positively stated items, and Items 1, 2, 3, 4, 11, 12, and 14 are the negatively stated items. A higher score indicates a higher level of stress. Scores higher than the middle of the range were considered as the presence of stress.

The insomnia severity index (ISI) consists of 7 questions that evaluate insomnia based on the International Classification of Sleep Disorders criteria. Each question is answered based on a five‐point Likert scale (None = 0, Mild = 1, Moderate = 2, Severe = 3, Very Severe = 4), with a total score ranging from 0 to 28. The total score is described as follows: no clinically significant insomnia (0–7), subthreshold insomnia (8–14), clinical insomnia (moderate severity) (15–21), and clinical insomnia (severe) (22–28).

All data were analyzed using SPSS version 24 (the IBM company). The Kolmogorov–Smirnov statistical test was used to evaluate the normality of data distribution. Mean and standard deviation were obtained for all parametric variables. Similarly, frequencies and percentages were derived for all nonparametric variables. The association between POH and other variables was tested using the chi‐square test. Also, the association between POH and some variables was analyzed using the linear regression. We carefully selected independent variables to reduce multicollinearity and ensure accurate results. Strongly intercorrelated variables were excluded, and interaction terms were added only when supported by biological or clinical evidence. The significance level considered was *p* ≤ 5%.

All included patients agreed to participate in the study by signing an informed consent form. The research project was approved by the institution's research ethics committee (IR.SSU.MEDICINE.REC.1400.457).

## 3 Result

A total of 116 people, 61.2% females, presenting with POH were evaluated. The mean age (± SD) of patients was 36.74 years (± 9.66). The mean age (± SD) at which patients reported noticing they had POH was 26.50 years (± 8.11). Regarding the physical examination, the majority of the population had moderate POH (47.4%), followed by mild POH (32.8%), severe POH (16.4%), and only 3.4% of the subjects had very severe POH. In many subjects, a positive history of multiple risk factors related to POH was noticed.

Table 1 presents the distribution of the subjects based on their demographic characteristics and association with the severity of POH. Statistical analysis showed that although women were more affected than men by POH, the difference was insignificant. The table notes that there was a significant relationship between a positive family history and the severity of POH. In contrast, a positive personal history of atopy was ineffective in the severity of POH.

**TABLE 1:** The association between demographic characteristics and the severity of POH among study population.

Variables		Severity of POH				All (*n* = 116)	*p*
		Mild (*n* = 38)	Moderate (*n* = 55)	Severe (*n* = 19)	Very severe (*n* = 4)		
Gender	Male	15 (39.5)	24 (43.6)	6 (31.6)	0 (0.0)	45 (38.8)	0.320
	Female	23 (60.5)	31 (56.4)	13 (68.4)	4 (100.0)	71 (61.2)	
Positive family history		10 (8.6)	32 (27.6)	11 (9.5)	4 (3.4)	57 (49.1)	0.002
Positive atopic history		5 (4.3)	25 (21.6)	11 (9.5)	3 (2.6)	44 (37.9)	0.432

*Note:* Values are presented as number (%). *p* < 0.05 is significant.

Abbreviation: POH, periorbital hyperpigmentation.

Table 2 indicates the distribution of POH severity in the study population. The number of subjects in each class and the percentage of total subjects in that category are indicated. Usage of hormonal pills and OCP was seen in 13.8% of the population, which is significantly related to the severity of POH.

**TABLE 2:** The association between different risk factors and the severity of POH among the study population.

Risk factors		Severity of POH				All (*n* = 116)	*p*
		Mild (*n* = 38)	Moderate (*n* = 55)	Severe (*n* = 19)	Very severe (*n* = 4)		
Hormonal pills and OCP		1 (0.9)	8 (6.9)	6 (5.2)	1 (0.9)	16 (13.8)	0.023
Exposure to the sun > 5 h a day		8 (6.9)	27 (23.3)	5 (4.3)	1 (0.9)	41 (35.3)	0.032
Habit of rubbing eyes		6 (5.2)	28 (24.1)	10 (8.6)	3 (2.6)	47 (40.5)	0.002
Daily usage of makeup substances		10 (8.6)	10 (8.6)	2 (1.7)	0 (0.0)	22 (19.0)	0.365
Computer and mobile > 5 h a day		25 (21.6)	34 (29.3)	11 (9.5)	3 (2.6)	73 (62.9)	0.892
Prolonged exposure to the television		1 (0.9)	8 (6.9)	6 (5.2)	2 (1.7)	17 (14.7)	0.005
Usage of prescription		12 (10.3)	21 (18.1)	9 (7.8)	3 (2.6)	45 (38.8)	0.303
Usage of sunglasses		25 (21.6)	22 (19.0)	9 (7.8)	1 (0.9)	57 (49.1)	0.072
Stress		14 (12.1)	29 (25.0)	15 (12.9)	2 (1.7)	60 (51.7)	0.072
Sleep quality	Absence of insomnia	9 (23.7)	12 (35.3)	2 (10.5)	0 (0.0)	23 (19.8)	0.681
	Subthreshold insomnia	18 (47.4)	2 (5.9)	8 (42.0)	2 (50.0)	51 (44.0)	
	Moderate insomnia	11 (23.9)	17 (50.0)	7 (36.8)	2 (50.0)	37 (31.9)	
	Severe insomnia	0 (0.0)	3 (8.8)	2 (10.5)	0 (0.0)	5 (4.3)	
Anemia		7 (6.0)	18 (15.5)	12 (10.3)	3 (2.6)	40 (34.5)	0.003
Diabetes		0 (0.0)	7 (6.0)	8 (6.9)	1 (0.9)	16 (13.8)	< 0.001
Hypertension		2 (1.7)	5 (4.3)	1 (0.9)	2 (1.7)	10 (8.6)	0.023
Thyroid disorders		2 (1.7)	12 (10.3)	4 (3.4)	3 (2.6)	21 (18.1)	0.004
Other underlying diseases		4 (3.4)	10 (8.6)	2 (1.7)	1 (0.9)	17 (14.7)	0.645

*Note:* Values are presented as number (%). *p* < 0.05 is statically significant.

Abbreviations: OCP, oral contraceptive pills; POH, periorbital hyperpigmentation.

A history of excessive sun exposure was given by 41 patients, which indicates the worsening of POH by sun exposure more than 5 h a day. Almost half of the total population expressed the habit of regularly wearing sunglasses. However, there was no significant relationship between the usage of sunglasses and the severity of POH. There was no relationship between the severity of POH and prolonged exposure (> 5 h a day) to bright screens such as computers and mobile phones; however, watching TV for more than 5 h a day played a key role in the severity of POH.

The habit of rubbing eyes was present in 40.5% of the population, significantly associated with the severity of POH. Among the various habits that were evaluated, daily usage of makeup substances and wearing prescriptions were found to have no relationship with the severity of this condition.

The mean score of participants in the PSS and ISI was 32.11 out of 56 and 12.58 out of 28, respectively. Over half of the evaluated patients considered their lives stressful. However, according to the ISI questionnaire filled out by participants, there was no relationship between sleep quality and the severity of POH.

A considerable amount of the population gave a history of associated systemic diseases that POH began or was aggravated by them. Positive history of anemia, diabetes, hypertension, and thyroid disorders was reported in 40,16,10, and 21 of the participants, respectively. However, there was no significant relationship between other underlying diseases and the severity of POH.

**GRAPH 1:**
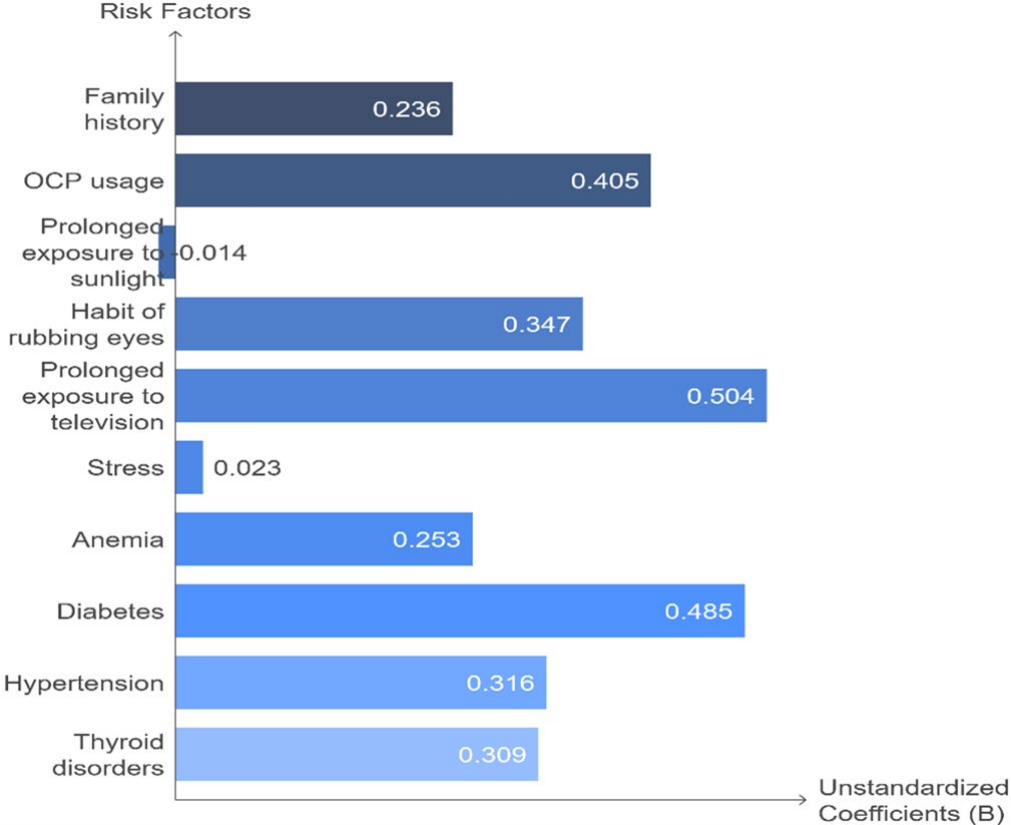
The association between different risk factors and POH based on linear regression analysis.

The results of the linear regression analysis can be seen in Table 3 and Graph 1. From this data, it is apparent that positive family history, usage of OCP, prolonged exposure to sunlight, the habit of rubbing eyes, stress, having diabetes, and thyroid disorders significantly affect POH. Also, it has been shown that the habit of rubbing eyes and prolonged exposure to television have more influence than other variables.

**TABLE 3:** The association between different risk factors and POH based on linear regression analysis.

Variables		Unstandardized confidents B	Standardized confidents (Beta)	*t*	*p*
Family history		0.236	0.0150	1.998	0.048
OCP usage		0.405	0.0178	2.347	0.021
Prolonged exposure to sunlight		−0.014	−0.009	−0.113	0.910
Habit of rubbing eyes		0.347	0.216	2.847	0.005
Prolonged exposure to television		0.504	0.226	2.998	0.003
Stress		0.023	0.201	2.745	0.007
Underlying diseases	Anemia	0.253	0.153	1.972	0.051
	Diabetes	0.485	0.212	2.788	0.006
	Hypertension	0.316	0.113	1.520	0.132
	Thyroid disorders	0.309	0.151	2.015	0.046

*Note:* *p* < 0.05 is significant.

Abbreviations: OCP, oral contraceptive pills; POH, periorbital hyperpigmentation.

## 4 Discussion

POH is a complex and unpleasing condition that results in an exhausted appearance [14]. Although this condition is not considered a medical concern, it creates a cosmetological problem, especially among women, which could be a nuisance, particularly for social development since adolescence [13]. It is important to make a detailed assessment of POH etiology so appropriate prevention and proper treatments can be prescribed to reduce this unpleasing condition.

The findings of the current study on the prevalence and risk factors of POH are consistent with several global studies while also highlighting unique aspects of our population. For instance, the association between positive family history and POH has been consistently reported in studies from Brazil and the UK [8, 15]. Additionally, a study conducted in India revealed that prolonged television watching, anemia, and irregular menstruation were significantly associated with POH, which aligns with this study. These global studies underscore the multifactorial nature of POH [12].

In the present work, patients were selected according to the presence of POH. To clarify the etiology of POH, patients were classified as mild in 32.8%, moderate in 47.4%, severe in 16.4%, and very severe in 3.4% of the individuals.

The mean age of patients was 36.74 years (9.66 SD). Other studies reported that adolescence was the most common age group affected by POH [12, 13]. In addition, the mean age at which patients reported noticing they had POH was 26.50 years (8.11 SD). This finding exactly seems similar to other studies [15]. As Matsui et al. reported, the onset of POH was 24 years. Also, all subjects of their study claimed that once POH was revealed, it did not vary [8].

Although women seemed to be more affected by POH than men, the difference was not reported to be significant. Also, Ranu et al. reported gender as an insignificant variable [15]. Some investigations have shown superiority in women as compared to men [1, 6]. According to various studies, the reason is that women are more concerned about their appearance and are inclined to visit a physician [16, 17].

The current study agrees with other investigations about the significant association between hormonal factors and the severity of POH. For instance, Chatterjee et al. reported a worsening of dark circles during pregnancy [1, 18, 19]. Numerous studies have revealed the role of menstrual irregularities and hormonal pills in POH [5, 20]. It seems that the incidence of POH will be increased in a society of women who are in the habit of taking OCP.

According to statistical analysis, a positive family history was associated with the severity of POH. Numerous studies have reported a dominant autosomal inheritance of POH, drawing attention to the variability in gene expression that can occur [1, 21, 22]. A study indicates that family history is the most significant etiology for POH [8].

In agreement with most studies, there was no significant relationship between atopy and POH [1, 21]. Matsui et al. have reported a significant effect of asthma but not allergy [8]. An association between atopic dermatitis and POH has been shown by Souza et al., and they expressed the habit of rubbing eyes in atopic patients as its reason [23]. Interestingly, the history of itching over the periorbital region has been reported as a significant risk factor in the current study. In numerous studies, excessive itching of the periorbital area in some conditions like contact dermatitis and atopic dermatitis has shown a significant relationship with POH [1, 4, 12, 16].

Sun exposure is one of the variables significantly related to POH. The association between this risk factor and POH is reported by Chatterjee et al., and some occupations like students, guards, and homemakers are mentioned, which creates excessive sun exposure [1]. Regarding prolonged exposure to bright screens, POH seemed to be significantly related to watching TV for more than 5 h a day but not using a mobile phone, consistent with another study [12].

Unsurprisingly, some life habits may be related to the severity of POH, among them stress [13]. Jage and Mahajan explained that stress causes the hypothalamus, pituitary, and adrenal axis (HPA axis) to release more melanocyte‐stimulating hormones, leading to hyperpigmentation [2]. The valid questionnaire that was used in the current study indicates this well.

It has been explained in other studies that the skin's HPA axis mimics the central HPA axis. Molecules like corticotropin‐releasing hormone and urocortin stimulate the production of peptides, which regulate local cortisol production and reduce inflammation. Stress affects this system by altering some cytokines and enzymes, affecting melanogenesis and skin homeostasis [24].

It would be interesting to compare POH across sleep behavior. The current study agrees with other studies that there was no association between sleep quality and POH [1, 13, 15]. However, a few investigations have reported inadequate sleep associated with POH [12]. Nevertheless, this is often the theory given by patients when asked about the etiology of their POH [1, 10]. In addition, a cultural bias in some countries associates exhaustion of the eyes with POH [1]. Sleep quality was not adequately examined by previous studies, so the ISI has been used in the current investigation to clear all the doubts.

Some underlying diseases were evaluated, among other risk factors. The positive history of anemia is related to POH. Due to selective vasoconstriction in the skin and low hemoglobin, there is inadequate oxygen supply in the periorbital region, which makes it look darker. In addition, facial pallor caused by anemia can make the area around eyes appear much darker [5, 16]. Few studies have been done about other diseases that can lead to POH. As far as we know, this is the only study that has discovered the association between hypertension, diabetes, and thyroid disorders with POH.

Interestingly, daily usage of cosmetic products was not a risk factor. This finding is consistent with the results of another investigation [1]. However, Mendiratta et al. found a history of some eye cosmetic products in a significant number of subjects [16]. In addition, there was no significant difference in patients who used prescriptions or not. Nevertheless, this isn't consistent with a few studies [12]. This study also reported sunglasses unrelated to POH, which was not evaluated by other studies.

## 5 Limitation

One of the limitations of this study is the lack of a control group, which makes it difficult to compare the results with a baseline. Future studies should include a control group to provide a more robust comparison and strengthen the validity of the findings.

*In conclusion*, POH is an undesirable condition that can lead to a decrease in life quality. The valid questionnaire in this study elucidates the insignificant relationship between sleep quality and POH, contrary to lay public opinion. This research indicates that POH is most likely to have a familial origin and unlikely to be caused by atopy. The main risk factors related to POH were the habit of rubbing eyes, exposure to television or sun for more than 5 h a day, and usage of hormonal pills. Some underlying diseases that seem to be related to POH are anemia, diabetes, hypertension, and thyroid disorders.

## Author Contributions

Conceptualization and writing, Fateme Heidari; formal analysis, Nasim Namiranian; supervision and physical examiner, Mohammad Ebrahim Zadeh; data collecting, Maryam Haji Abol Zadeh. All authors have read and agreed to the published version of the manuscript.

## Ethics Statement

The authors confirm that the ethical policies of the journal, as noted on the journal's author guidelines page, have been adhered to and the appropriate ethical review committee approval has been received. (IR.SSU.MEDICINE.REC.1400.457).

## Consent

Informed consent was obtained from all patients involved in this study.

## Conflicts of Interest

The authors declare no conflicts of interest.

## Data Availability

The data that support the findings of this study are available on request from the corresponding author. The data are not publicly available due to privacy or ethical restrictions.
