# Correction to “The frequency of periorbital hyperpigmentation risk factors”

**DOI:** 10.1111/jocd.70279

**Published:** 2025-06-19

Citation to article being corrected:

Fateme Heidari, Mohammad Ebrahim Zadeh, Maryam Haj Abol Zadeh, Nasim Namiranian. The frequency of periorbital hyperpigmentation risk factors. Journal of Cosmetic Dermatology. 2025. DOI: 10.1111/jocd.70036

Description of error:

In the originally published version of the manuscript, the order of the authors was incorrectly listed as:

1. Fateme Heidari

2. Mohammad Ebrahim Zadeh

3. Maryam Haj Abolzadeh

4. Nasim Nemiranian

The correct order of the authors should be:

1. Mohammad Ebrahimzadeh Ardakani

2. Fateme Heidari (Corresponding Author)

3. Maryam Haji Abolzadeh

4. Nasim Nemiranian

We apologize for this error and respectfully request the correction of the author order in the published version. The corresponding author remains Fateme Heidari.

We are grateful for your understanding and assistance in implementing this correction.

